# The filamentous fungal pellet—relationship between morphology and productivity

**DOI:** 10.1007/s00253-018-8818-7

**Published:** 2018-02-22

**Authors:** Lukas Veiter, Vignesh Rajamanickam, Christoph Herwig

**Affiliations:** 1 Research Area Biochemical Engineering, Institute of Chemical Engineering, TU Wien, Gumpendorfer Straße 1a, 1060 Vienna, Austria; 2 Christian Doppler Laboratory for Mechanistic and Physiological Methods for Improved Bioprocesses, TU Wien, Gumpendorfer Straße 1a, 1060 Vienna, Austria

**Keywords:** Fungal pellet morphology, Interlinks between productivity and morphology, Variability and alteration of morphology, Analysis of morphology

## Abstract

Filamentous fungi are used for the production of a multitude of highly relevant biotechnological products like citric acid and penicillin. In submerged culture, fungi can either grow in dispersed form or as spherical pellets consisting of aggregated hyphal structures. Pellet morphology, process control and productivity are highly interlinked. On the one hand, process control in a bioreactor usually demands for compact and small pellets due to rheological issues. On the other hand, optimal productivity might be associated with less dense and larger morphology. Over the years, several publications have dealt with aforementioned relations within the confines of specific organisms and products. However, contributions which evaluate such interlinkages across several fungal species are scarce. For this purpose, we are looking into methods to manipulate fungal pellet morphology in relation to individual species and products. This review attempts to address (i) how variability of pellet morphology can be assessed and (ii) how morphology is linked to productivity. Firstly, the mechanism of pellet formation is outlined. Subsequently, the description and analysis of morphological variations are discussed to finally establish interlinkages between productivity, performance and morphology across different fungal species.

## Introduction

In submerged culture, filamentous fungi either grow in spherical pellets, consisting of compact hyphal aggregation, or in filamentous form, featuring homogeneously dispersed hyphae (Pirt 1966). A pellet forming cultivation system is necessarily heterogenous and aerobic (Wosten et al. 2013; Amanullah et al. 2001). The morphological state of filamentous fungi has a large impact on process performance in a bioreactor. Morphology, physiology and productivity of filamentous fungi are influenced by process parameters on many levels (Ehgartner 2017). These characteristics are highly interlinked with each other and therefore have to be addressed collectively to understand the interdependencies between them. For example, in free mycelia, high biomass concentrations result in highly viscous fermentation media, resulting in issues with gas−liquid mass transfer, liquid mixing and a generally complex rheology in *Aspergillus terreus* (Porcel et al. 2005). However, pellet morphology also comes with disadvantages: within *Penicillium chrysogenum* pellets, problems with internal transport of substrates and products may occur, depending on size and compactness of pellets (Dynesen and Nielsen 2003). Therefore, it is highly important to individually assess each production task.

This review strives to provide a general overview across several pellet forming fungal species. In the following, basic forces that trigger pellet formation are briefly summarised. Subsequently, description and analysis of pellet morphology are outlined. This provides the basis for understanding the interlinks between productivity, performance and morphology, as will be discussed in the final chapter of this review.

## Mechanism of pellet formation

Traditionally, fungal pellets are attributed to either coagulative or non-coagulative types of formation (Nielsen et al. 1995; Pirt 1966). Table 1 provides an overview on pellet classification across several species.

**Table 1:** Overview on several fungal species: agglomeration type, variations in pellet morphology and possibilities of morphological alteration; if not mentioned otherwise, preferred morphology refers to cultivation

Type	Species	Preferred morphology	Alteration of morphology	References
Coagulative type	*Aspergillus* *A. niger* *A. nidulans* *A. oryzae*	For citric acid production: Swollen hyphal branches, compact agglomerates = clumps, pellets featuring thin biomass layers and loose core For production of fructosyl-transferase: Small, spherical	Strong agitation (filament fragmentation wanted) Aeration using oxygen/air 1:1 mixture, high growth rate low pH (2.0 ± 0.2), Manganese presence Spore inoculum level Pellet dispersion instead of spore inoculum Surfactant: Tween 20	Wilkinson 1998 Papagianni 2007 Papagianni and Mattey 2006 Prosser and Tough 1991 Wang et al. 2017 Kurakake et al. 2017
	*Phanerochaete chrysosporium*	Small pellet size (~ 5.5 mm^3^) for lignin peroxidase production	High shear rate	Zhang and Zhang 2016 Zmak et al. 2006
	*Blakeslea* and *Choanephora*	Compact pellet form	Anionic polymers hinder spore aggregation prior to germination	Prosser and Tough 1991
	*C. unicolor*	Compact star-shaped pellet form,	Microparticle-enhanced cultivation (Al_2_O_3_ particles)	Antecka et al. 2016
	*C. fumago*	Compact pellet form	Small inoculum volume, carbon source (fructose)	Carmichael and Pickard 1989
	*C. sinensis*	Small and loose pellets	Surfactant: Tween 80 pH: optimum 6.0	Liu and Wu 2012
Non – coagulative type	*A. ochraceus*	Compact pellet form	Spore inoculum and agitation	Abd-Elsalam 2009
	*R. oryzae*	Loose pellets in lactic acid production	Medium: peptone, dextrose, calcium carbonate	Liao et al. 2007
Hyphal element agglomerating type	*P. chrysogenum*	“Fluffy” pellet to ensure largest possible active layer with high quantities of cytosol	Aeration using oxygen/air, controlled growth rate CSL in medium, CO_2_ concentration Spore inoculum level Physiological process control based on morphological modelling approach	Wilkinson 1998 Nielsen et al. 1995 Prosser and Tough 1991 Ho and Smith 1986 Posch and Herwig (2014)
	*P.* sp. L1	Large pellet size	Pellet dispersion instead of spore inoculum	Liu et al. 2017

**Fig. 1:**
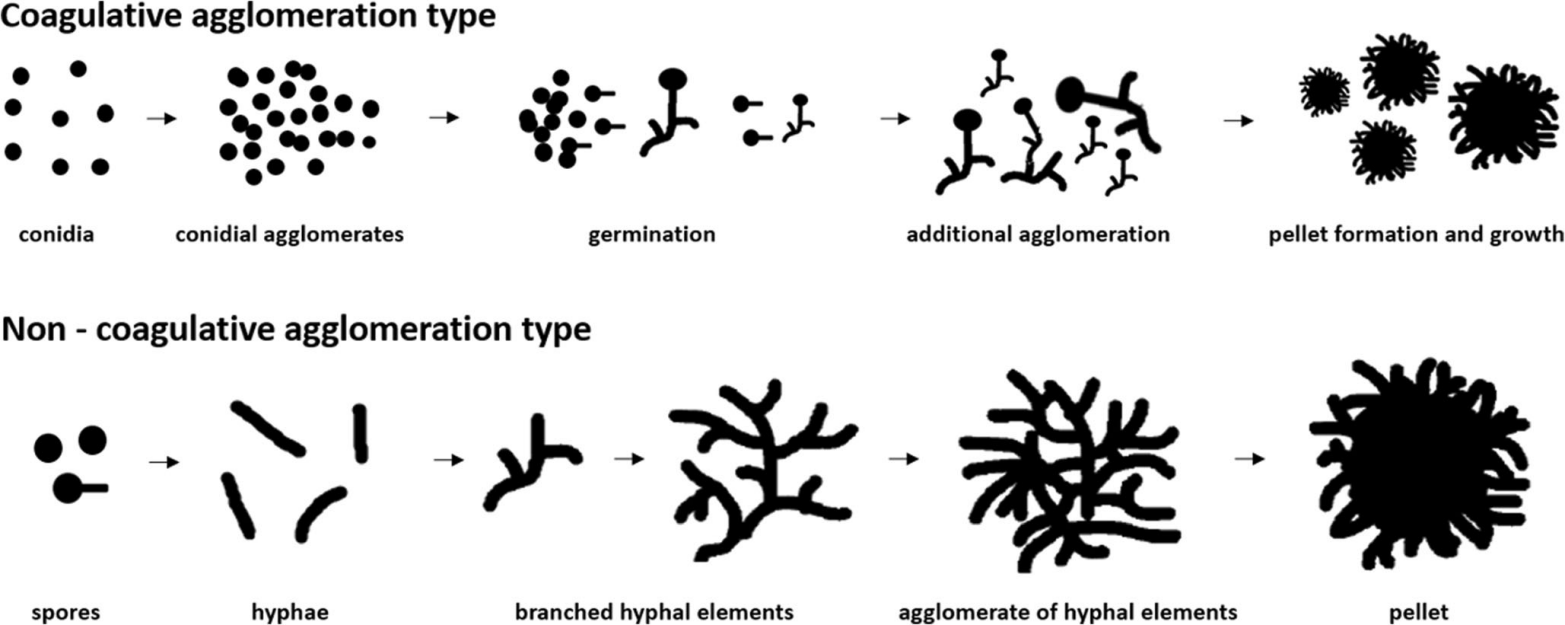
Illustration depicting the coagulative and non-coagulative model of pellet formation

Figure 1 depicts the coagulative and non-coagulative model of pellet formation. In the coagulative type, spores aggregate fast and subsequently germinate involving hyphal tip growth (Zhang and Zhang 2016). Finally, a great number of spores of the coagulative type form pellets. On the contrary, spores of the non-coagulative type germinate before pellet formation. Therefore, one pellet theoretically can be formed by one single spore. Non-coagulative pellet formation is interlinked with agitation and aeration (Pazouki and Panda 2000). It should be noted, however, that depending on cultivation factors, fungal species will exhibit different morphological behaviour. Therefore, a final classification of coagulation type is difficult (Zhang and Zhang 2016; Pazouki and Panda 2000). For instance, *P. chrysogenum* exhibits characteristics of both types, as agglomeration of hyphal elements leads to hyphal clumps which form pellets in the end (Wilkinson 1998; Nielsen et al. 1995). Wilkinson (1998) therefore suggested a new term for species like *P. chrysogenum*: the hyphal element agglomerating type.

Electrostatics, hydrophobicity and interactions between spore wall components are main triggers for pellet formation (Zhang and Zhang 2016). Fungal spores generally exhibit negative surface charges (Douglas et al. 1959) which are affected by pH and ionic strength (Akiba et al. 1994). In a simplified view, higher pH values are considered to cause negative charges which in turn decrease spore aggregation (Zhang and Zhang 2016). However, conflicting observations suggest that electric repulsion is not the only driving factor. For *Aspergillus niger*, it was proposed that mainly single conidia are affected by surface charge, aggregated conidia might additionally be stabilised through higher electric charges (Grimm et al. 2005b). Wargenau et al. (2011) further found that the electrostatic surface potential of *A. niger* spores is considerably affected by pH-dependent release of melanin pigment leading to an irreversible reduction of the outermost layer between spores and surrounding solution. In short, they concluded that thickness and accessibility of surface coating, as well as ionic strength of the medium have additional effects on pH-dependent spore repulsion. Furthermore, pH also heavily affects hydrophobicity of proteins (Pascual et al. 2000). Especially hydrophobins strongly influence adhesion forces. For instance, deletion in different hydrophobin encoding genes in *A. nidulans* mutants leads to a decrease in pellet biomass and size (Dynesen and Nielsen 2003).

Another important contribution to pellet formation are interactions between spore wall components, notably salt bridging between polysaccharides (Zhang and Zhang 2016). Gerin et al. (1993) even stated that aggregation is only dependent on salt bridging between polysaccharides in *Phanerochaete chrysosporium*. Depending on respective physiological conditions, spores undergo several changes in spore wall components and properties (Zhang and Zhang 2016). In the beginning, water uptake combined with the swelling of spores leads to an increase of metabolic activities that leads to water uptake combined with swelling of spores. This is followed by germination of conidia, which represents the beginning of fungal growth. After germination, hyphal elongation takes place and the fungus can initiate hyphal branching which is critical for the formation of the mycelium (Paul and Thomas 1996). These initial steps in fungal development strongly affect spore aggregation: shortly after incubation, spore aggregation occurs due to electrostatic and hydrophobic interactions. However, as soon as the swelling of spores leads to polysaccharides being exposed, hydrophobic interactions decrease in favour of interactions between cell wall components (Priegnitz et al. 2012; Zhang and Zhang 2016).

Recently, efforts to describe aggregation kinetics via population dynamics modelling were made (Grimm et al. 2004; Lin et al. 2008). Via an in-line particle size analyser, two distinct aggregation steps of coagulating fungi *A. niger* could be described. In the first step, conidial aggregates are formed from individual conidia. Population dynamics in this first step combine formation and disappearance of particles through aggregation and breakage. The second step considers germination of conidia, thereby hyphal growth greatly increases the surface area necessary for aggregation. This process is closely related to the specific length growth rate and the rate of germination (Grimm et al. 2005b). More recently, Priegnitz et al. (2012) concluded that germination of conidia within the second step is essential for pellet formation in *A. niger*.

All of these findings signify that specific aggregation triggers vary during the pellet formation process. When summarising influences on electrostatic and hydrophobic interactions, it can be concluded that pH is the driving factor for both. Regarding interactions between spore wall components, no definite conclusions can be drawn from the scientific literature. However, publications suggest that it is mostly driven by the presence of polysaccharides (Zhang and Zhang 2016).

## Description and analysis of pellet morphology

**Fig. 2:**
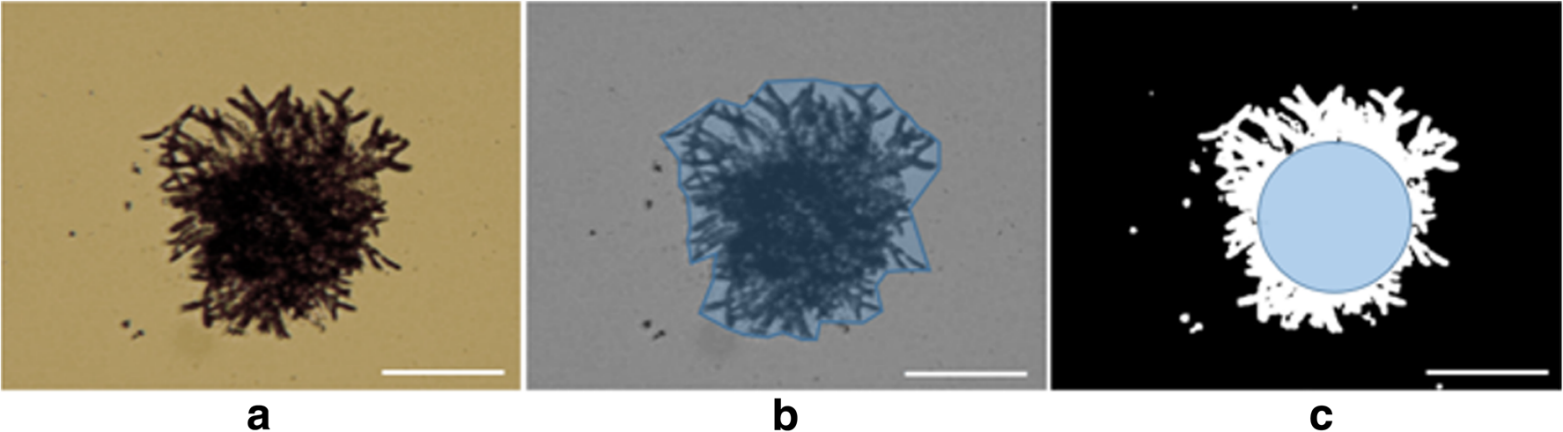
Light microscopic images of the same *P. chrysogenum* pellet, white line = 100 μm; parameters for depiction of core region and hairy region. Blue line (**b**): perimeter for estimation of roughness, blue area (**b**): convex area for fullness calculation, blue circle (**c**): core area

Pellet diameters extend from a few hundred micrometres up to 1 mm. The basic morphology of fungal pellets is characterised by the compact inner core consisting of densely packed hyphae (Cox et al. 1998). The existence of such a core is the distinction between pellets and clumps. The core is surrounded by a less dense hyphal layer, known as the hairy region (Krull et al. 2010). Core region and hairy region can be defined by parameters, mostly derived from microscopic analysis as displayed in Table 2 (Paul and Thomas 1998; Posch et al. 2012; Ehgartner 2017). Figure 2 displays images related to respective parameter estimation.

**Table 2:** Parameters for pellet characterisation

	Parameter	Definition
Core region	Fullness	Ratio of actual area of the particle to convex area; = 1 for pellets without hairy regions
	Circularity	Deviation from a true circle, derived from area and perimeter
	Core area	Encompasses core equivalent diameter
Hairy region	Roughness	Irregularity of the perimeter of an object, obtained from circularity measurement around an object boundary
	Equivalent diameter	Diameter of a circle having the same area as the pellet

The most common method to analyse pellet morphology is microscopy (Cox et al. 1998; Paul and Thomas 1998; Posch et al. 2012). Usually, image recording is combined with some form of automated image analysis software to ensure statistical verification. Thereby most parameters described in Table 2 can be determined. However, sample preparation on microscopy slides is problematic as it affects fungal biomass dimensions due to potential squeezing between cover slide and specimen slide. In this process also, all three dimensional informations are lost.

Confocal laser scanning microscopy (CLSM) in combination with staining is a powerful method for visualisation of metabolically active regions in pellets. Furthermore, biomass segregation, density and growth could by monitored via this technique (Villena et al. 2010). Wargenau and Kwade (2010) were able to directly measure *A. niger* spore adhesion forces at different pH and ionic strength values by further employing atomic force microscopy (AFM). Information on hydrophobic and hydrophilic domains can be obtained via adhesion force mapping across spore surfaces. This technique also provides information on the role of hydrophobins on adhesion (Krull et al. 2013). Via scanning electron microscopy (SEM), Villena and Gutierrez-Correa (2007) were able to differentiate highly intertwined superficial hyphae and densely packed deep mycelium in *A. niger* pellets.

In recent years, flow cytometry in combination with fluorescent staining (Golabgir et al. 2015) was also used to depict morphological distributions in filamentous fungi samples. Limitations of this technique are also related to large pellet dimensions; consequently, flow cytometers must be adapted for large particle size ranges. Flow cytometry is very fast and statistically robust as a large number of particles can be measured in low time spans. When compared with microscopic image analysis, Ehgartner et al. were able to obtain similar size parameters as well as enhanced characterisation of fungal pellet compactness and hyphal region (Ehgartner et al. 2017). Potential issues include size exclusion effects at the sampling tube. Rather large pellet dimensions might lead to overrepresentation of smaller particles. In addition, large particles could cause saturation effects on certain detector signals (Ehgartner 2017). Thereby important information about various morphological features (e.g. hairiness/fluffiness) is lost in relation to pellet diameters.

Other methods successfully used for macroscopic analysis of *A. niger* include focused beam reflectance measurement (FBRM), a size-determining optical method based upon backscattering laser light (Krull et al. 2013). Grimm et al. (2004) characterised conidial inocula to determine effects on the conidial aggregation process. Lin et al. (2010) analysed pellet slices of several pellet regions via microscopy in order to determine regularity/circularity and surface structures. Furthermore, the impact of pellet surface structure on sedimentation behaviour in water was described. Although optical methods involving microscopy and flow analysis can be used to determine macro-morphological features, characterisation of pellet morphology and physiology remains challenging. This is mainly due to the fact that common macro-morphological parameters do not cover all important pellet characteristics (Hille et al. 2006). Regarding physiology, diffusion of substrates into pellets needs to be considered dependent on varying degrees of compactness and roughness, which cause variable oxygen uptake rates (Krull et al. 2010). If homogeneous biomass distribution is assumed, oxygen concentration gradients are the same for pellets of equal size. However, while mean biomass density may be similar for pellets from different cultivations, their inner structures could differ considerably. Hille et al. (2006) describe variations in biomass density between *A. niger* pellets related to inoculum conditions. The authors used CLSM to measure fluctuating density distributions in the outer layers. Dense outer pellet sections strongly impact oxygen delivery and consumption in two ways: (i) oxygen consumption is increased and (ii) substrate diffusion is restricted. Furthermore, hydrodynamics significantly affect substrate diffusion and internal oxygen concentration profiles. In their periphery, loosely structured pellets exhibit convective tendencies in substrate transport, whereas in tightly packed core regions, diffusion is predominant. Assuming that the number of hyphal tips is the essential factor for oxygen conversion, dense pellets are favoured despite restricted oxygen transport (Hille et al. 2005). Related oxygen concentration profiles in fungal pellets obtained via microelectrodes were successfully correlated with hyphal distribution in outer pellet regions (Grimm et al. 2005b).

Consequently, a major task when characterising morphology is to come up with novel or expanded parameters also depicting micro-morphological and physiological characteristics. Such parameters must come from robust analytical tools and have to consider culture conditions as well, thereby deepening understanding of fungal morphology and physiology.

## Effects of pellet morphology on productivity

There have been several attempts to specify definite links between morphology and productivity (Walisko et al. 2015), but no simple relationship that would favour a specific morphology has been identified. Grimm et al. (2005a) identified the large amount of process parameters affecting morphology as an important factor. Consequently, any reported co-dependencies are limited to individual processes with specific organisms and products. When comparing these various results, it becomes clear that there are no generally applicable rules. In fact, different morphologies are in favour of different products, conflicting reports are available even for the same species. Consequently, comprehensive process design must align morphology with metabolism and productivity (Grimm et al. 2005a). For fungal pellets, the following relationship to metabolism has been proposed (Grimm et al. 2005a): biomass density is inhomogeneous and inversely proportional to pellet porosity, which in turn leads to limited accessibility for nutrients. Such complex hyphal networks hinder substrate uptake thereby directly affecting metabolism. Within this context, oxygen has been identified as the prime limiting substrate.

Interlinkages between pelletised morphology and productivity were studied intensively for *A. niger* (Papagianni 2007). While it is not entirely clear if pelletised or filamentous morphology is more appropriate for citric acid production, it has been proven that productivity is linked to short, swollen hyphal branches that may have swollen tips. Available literature (Papagianni 2007; Papagianni and Mattey 2006; Zhang and Zhang 2016) mainly favours compact agglomerates and pellets (< 0.5 mm), but conflicting reports also find pellet growth disadvantageous. More detailed studies (Papagianni et al. 1998) have suggested that the so-called clump form would be most suitable. Such clumps are stable agglomerates of filaments, but do not exhibit a compact core (Papagianni 2007) as opposed to pellets. Driouch et al. (2012) were able to stir *A. niger* pellet morphology towards a reduced thickness of biomass layer via smaller pellets as well as altered core shell structure to enhance productivity. Such a trend was also established for *A. terreus* as smaller pellets are deemed more compact, hence more stable (Porcel et al. 2005). However, for *A. oryzae*, filamentous growth achieved higher α-amylase productivity (Carlsen et al. 1996).

For *P. chrysogenum*, Paul and Thomas (1998) discriminated between different parts of hyphae: actively growing regions, non-growing cytoplasm, vacuolised hyphae and inactive regions (Justen et al. 1998). Penicillin production is happening in the non-growing cytoplasm (equivalent to subapical hyphal cells). This differentiation can be expanded to larger pellet structures: also pellets feature distinct active regions (Baumgartl et al. 1984). Such an active region is found in the outer layer of the pellet, which contains high quantities of cytoplasm. Cytoplasm content is decreasing in the inner regions. The pellet’s centre exhibits hyphal degradation (Ehgartner 2017). Therefore, the largest possible active layer would characterise the optimal pellet form for Penicillin production in bioreactors.

Pellet formation greatly enhances lactic acid production and fumaric acid when compared to clump-like morphological structures for *R. oryzae* (Liao et al. 2007). This is especially interesting as less compact morphology usually displays more productivity as viability is apparently not hindered by diffusion through dense structures. Fu et al. (2014) also found that low-density pellets exhibiting a hollow core greatly decreased lactic acid production. However, pellet densities over 60 kg/m^3^ were identified to be disadvantageous as this led to a generally limited mass transfer.

Kim and Song (2009) found that pellet size of white rot fungus *Pleuratus ostreatus* affects its biodegrading capacity. The overall biodegradation rates were closely related to laccase and esterase activity. Small-pelleted cultures were determined as favourable morphology for optimal activity of both degradative enzymes.

In general, any enhanced productivity of mycelia and clumps is due to easier supply of oxygen and substrates. Consequently, the ideal pellet is a large agglomeration of productive sections that have open access to substrates and oxygen. As mentioned, such a loose structure is associated with highly viscous fermentation media, resulting in issues with gas−liquid mass transfer, liquid mixing and complex rheology. Therefore, morphological optimisation has to consider the following: rheological requirements for the bioprocess on the one hand and ideal pellet compactness for enhanced productivity on the other hand. Possible options to favourably alter pellet morphology are discussed below.

## Alteration of pellet morphology

In this section, we take a closer look at factors, which affect pellet formation and morphology. All these potential factors are interdependent. Therefore, any form of morphological control strategy is highly complex.

## Agitation

The general rule states that strong agitation results in smaller pellets. If pellet formation is proceeded by spore aggregation, strong agitation could decrease pellet growth (Prosser and Tough 1991). If pellets have already formed, they are affected by agitation in two ways (Tanaka et al. 1975): (1) hyphal elements on the pellet surface can be shaved off and (2) there is also the possibility of total pellet rupture. In general, the shaving of hyphal elements is preferred, as total pellet rupture would lead to impurity release.

For several *Aspergillus* species, strong agitation forces facilitate a morphology of short, thick and highly branched filaments advantageous for citric acid production (Papagianni 2007). Fragmentation of filaments can also occur, but is mainly limited to old and heavily vacuolated parts. Therefore, there is a leeway for beneficial breakage of filaments through agitation: a balance between new growth and fragmentation of inactive sections (Papagianni 2007).

*P. chrysogenum* also displays clear relations between morphology and agitation. Agglomeration of hyphal elements and thereby pellet formation is negatively affected by strong agitation (Walisko et al. 2015). Nielsen et al. (1995) have reported that a shift from pellet morphology to disperse mycelia could be achieved for fed-batch cultivations.

Pellet size of *P. ostreatus* is predominantly controlled via agitation; Kim and Song (2009) were able to obtain either large-pelleted or small-pelleted morphology at respective agitation speeds. Tinoco-Valencia et al. (2014) further studied agitation and aeration effects on *P. ostreatus* growth. They observed predominantly pellet morphology. High agitation in combination with high aeration flow rates led to increased oxygen mass transfer and decreased pellet size, respectively (Serrano-Carreon et al. 2015). Consequently, growth rate and maximum biomass concentration were increased.

To recapitulate, agitation considerably alters morphology, especially during agglomeration. If pellets have already formed, strong agitation could lead to adverse effects like pellet rupture. Several production processes favour thin biomass layers and loose core structures in pellets due to strong agitation, for example citric acid production in *A. niger* or lignin peroxidase production in *Phanerochaete chrysosporium.*

## Broth viscosity, medium composition and pH

In general, an inverse relation between pellet size and broth viscosity has been reported. For example, Prosser and Tough (1991) state that adjustment of broth viscosity via carbohydrates is advantageous for cultivations of *Blakeslea* and *Choanephora*. The use of anionic polymers has been shown to hinder spore aggregation prior to germination. Thereby organisms that usually favour pellet growth can be stirred towards dispersed growth and vice versa. Trinci (1983) reported this effect for *Aspergillus* and some basidiomycetes. These findings might also apply to naturally formed polysaccharides as has been speculated for *P. chrysogenum* (Prosser and Tough 1991).

Kisser et al. (1980) studied the effect of manganese sufficient or deficient cultivation on *A. niger* morphology and cell wall composition in citric acid production. Omission of manganese from the nutrient medium results in “abnormal morphological development which is characterised by increased spore swelling and bulbous hyphae”. Only compact pellets produce citric acid; consequently, manganese deficiency is to be avoided. The same effects of manganese deficiency have also been confirmed by Papagianni et al. (1998). Sensitivity to the presence of trace metals equals that there is also sensitivity for metal complexing ions like EDTA (Prosser and Tough 1991).

Wucherpfennig et al. (2011) studied the effect of osmolality on *A. niger* morphology and productivity. Culture broth osmolality was increased by the addition of sodium chloride. It was found that pellet size declined with osmolality. However, it was determined that the culture was also becoming more homogenous.

Investigations into optimal medium composition for *R. oryzae* implied that peptone had a positive effect on pellet formation. The addition of metal ions as well as interaction of metal ions and peptone impeded pellet formation (Liao et al. 2007). Through the concentrations of potato dextrose broth, soybean peptone and calcium carbonate in the medium pellet size were controlled. Fu et al. (2014) additionally state that pellet density is positively affected by addition of peptone. Under low peptone concentrations, low-density pellets with hollow structures were observed.

For *P. chrysogenum*, addition of corn steep liquor (CSL) to the culture medium is known to have positive effects on pellet formation at an early stage and penicillin production (Sajjad-Ur-Rahman et al. 2012). There is still ongoing research if the state-of-the-art penicillin fermentation medium (composed of CSL, glucose, lactose, minerals, oil, nitrogen source and precursor) can be modified and improved. Cultivation in buffered medium had positive effects on biomass growth for several *Penicillium* species: pellets displayed greater diameters as well as smaller hyphae on the surface (Walisko et al. 2015). Studies using SEM indicate that the morphology of *P. chrysogenum* is affected by CO_2_ presence in the medium: at low CO_2_ concentrations (up to 8%), the filamentous form was predominant, higher concentrations led to the formation of swollen and stunted hyphae affecting pellet morphology (Ho and Smith 1986).

As noted previously, pH is a driving factor on electrostatic and hydrophobic interactions. For citric acid production, pH of culture medium is preferably low and strongly affects production, simply because of the pH sensitivity of enzymes in the TCA cycle (Papagianni 2007). Morphological development of small pellet aggregates and short filaments is best sustained at pH values of 2.0 ± 0.2. Liu and Wu (2012) also found pH-related effects on the morphology of *C. sinensis*. The mycelial pellets became less uniform at lower pH (< 6.0). Filamentous growth was observed at higher pH (8–9). The growth of ascomycete fungus *Neurospora intermedia* in uniform pellet form can be achieved using a pH range of 3.0–4.0 (Nair et al. 2016).

In recent years, the effect of surfactants has been tested. Liu and Wu (2012) found that Tween exhibited a promoting effect on production of exopolysaccharide in the fungus *Cordyceps sinensis*. Improved productivity was combined with medium inhibited pellet formation leading to small and loose pellets. More recently, Kurukake et al. (2017) observed that *A. oryzae* pellets became mall and spherical on addition of Tween surfactant. Furthermore, production of fructosyl-transferase could be enhanced. Addition of Tween also led to an increased specific surface area of *Pleurotus eryngii* pellets (Wu et al. 2016). Antecka et al. (2016) successfully applied a morphological engineering technique to a laccase production process in basidiomycetes. The authors found that the addition of Al_2_O_3_ microparticles led to a decrease of pellet size, shape and structure in *Cerrena unicolor* and *Pleurotus sapidus*. Similar morphological engineering techniques—namely the use of magnesium silicate microparticles—were also successful for oleaginous fungus *Mortierella isabellina* (Gao et al. 2014).

To summarise, effects of medium composition on morphology and productivity are highly diversified and entirely dependent on species and process conditions. According to the available literature, we can only assume that pH has considerable impact across several species. Nevertheless, a selection of preferred medium compositions is available for several fungal pellet processes.

## Spore inoculum level and other inoculation strategies

Generally, an inverse relationship between spore number and pellet size has been identified for several *Aspergillus* species (Prosser and Tough 1991). Citric acid production in *A. niger* is particularly affected by spore inoculum. Papagianni and Mattey (2006) found studied spore development and morphology in a bioreactor in relation to spore inoculum concentrations. They classified four morphological classes: globular pellets, elongated pellets, clumps and free mycelia. Glucosamine formation and release was clearly related to spore inoculum level. At higher inoculum levels (10^8–10^9 spores/mL), lower dissolved oxygen levels were measured which led to mycelium developed in dispersed morphologies. For *Caldariomyces fumago*, aforementioned relationships were also found. Additionally, pellet density was reported to be inversely related to pellet size (Carmichael and Pickard 1989).

For *P. chrysogenum*, it was reported that with low spore inoculum concentrations, only few agglomerations of hyphal elements happen which in turn leads to pellets with small diameters (Nielsen et al. 1995). At very high concentrations (> 10^5 spores/mL), the hyphal element size is small and agglomeration, respectively, pellet formation does not occur in the first place. For a spore inoculum concentration of 3.7 * 10^4 spores/mL maximum, pellet concentration was measured which amounts to roughly 1.5 * 10^4 pellets/mL. In a different study, Posch and Herwig (2014) describe a positive effect of spore inoculum concentration on penicillin formation during early production phases, which feature excess substrate, biomass growth and overflow metabolism. Simultaneously, a negative effect on pellet morphology is observed: pellets develop larger structures at reduced spore inocula; consequently, pellet breakage resulting in dispersed morphology is more likely.

In recent years, the so-called pellet-dispersion strategy has brought promising results for seed cultivation of *A. niger niger* (Wang et al. 2017). Thereby pellets were used to substitute spores during inoculation. Traditionally, long time spans are needed for spore preparation of seed culture. This drawback was avoided through the development of a novel seed-recycling process. The morphological structure displayed “densely intertwined hyphae” and pellet compactness increased considerably. A 48-h pellet inoculum was also advantageous for growth of a newly isolated nitrifying fungus defined as *Penicillium* sp. L1. When compared to inoculation with spore suspension, pellet size could be increased significantly by using a pellet inoculum (Liu et al. 2017).

## Other factors—aeration and growth rate

*A. niger* generally features an indirect relation between pellet compactness and aeration rate. However, using a 1:1 oxygen/air mixture leads to dense growth and increased hyphal branching (Prosser and Tough 1991). Krull et al. (2013) state that *A. niger* pellets derived from high aeration rates have overall a much larger structure and have unstructured and irregular outer sections. Low aeration results in rather compact peripheral structures. By comparison, it was found that agitation effects are less severe.

Some *Aspergillus* species are associated with filamentous morphology at low growth rates and reversibly produce pellets at higher rates (Prosser and Tough 1991). High growth rates in *P. chrysogenum* cultivations lead to an increase in hyphal branching (Nielsen et al. 1995). Decreasing the specific growth rate was followed by pellet breakage. In addition, hyphal elements were torn away from the surface. Nielsen et al. assume that such pellet breakage happens due to cell lysis within the pellet, which leads to a loss in stability. Posch and Herwig (2014) were able to describe parameter effects on pellet morphology through morphological and physiological bioprocess modelling approaches. In the beginning, high growth rates yield large pellet fractions. If pellet growth reaches a critical level, a transition phase leads to dispersed growth due to increasing pellet erosion and breakage.

Summarising, factors such as agitation and media pH have universal impact on pellet morphology. However, the effect of most factors described in this section only applies to specific species and processes. In general, definite guidelines for morphological alteration or control cannot be given.

## Conclusions

Any preference in fungal pellet morphology depends on species and specific task at hand. To timely assess current process morphology, novel techniques like flow cytometry combined with fluorescent staining are convenient. Once desired relations between pellet form and productivity have been established, one can draw from a multitude of options to favourably alter and optimise morphology. Some of these possibilities are summarised in Table 1: in this table, pellet forming species are divided into coagulative or non-coagulative type. Based upon this distinction, preferred pellet morphologies are cited, extended by possible techniques to favourably alter morphology.

From our perspective, modelling approaches involving raw data from morphological classification seem promising to increase process understanding. We envision a combination of timely morphological assessment with specific morphological alteration in order to favourably stir fungal pellet processes towards increased productivity.
